# Supplementary material for: Experimental infection of cattle with Mycobacterium tuberculosis isolates shows the attenuation of the human tubercle bacillus for cattle
Source: Sci Rep. 2018 Jan 17;8:894. doi: 10.1038/s41598-017-18575-5 (PMC5772528; doi:10.1038/s41598-017-18575-5)
Supplement: Supplementary file 1 — Supplementary information [file 41598_2017_18575_MOESM1_ESM.pdf]

Experimental infection of cattle with *Mycobacterium tuberculosis* isolates shows the attenuation of the human tubercle bacillus for cattle

Bernardo Villarreal-Ramos[1], Stefan Berg[1], Adam Whelan[1,*], Sebastien Holbert[2,3], Florence Carreras[2,3], Francisco J. Salguero[4], Bhagwati L. Khatri[1], Kerri Malone[5], Kevin Rue-Albrecht[5,6,§], Ronan Shaughnessy[5], Alicia Smyth[5], Gobena Ameni[7], Abraham Aseffa[8], Pierre Sarradin[9], Nathalie Winter[2,3], Martin Vordermeier[1], Stephen V. Gordon[5,10,11,12,¶]

[1]Animal and Plant Health Agency, Weybridge, Surrey KT15 3NB, UK.
[2]Infectiologie et Santé Publique (ISP-311), INRA Centre Val de Loire, F-37380 Nouzilly, France
[3]Université de Tours, UMR 1282, Tours, 37000, France
[4]Department of Pathology and Infectious Diseases, School of Veterinary Medicine, University of Surrey, Guildford, UK
[5]UCD School of Veterinary Medicine, University College Dublin, Ireland.
[6]UCD School of Agriculture and Food Science, University College Dublin, Ireland.
[7]Aklilu Lemma Institute of Pathobiology, Addis Ababa University, PO Box 1176, Addis Ababa, Ethiopia.
[8]Armauer Hansen Research Institute, P O Box 1005, Addis Ababa, Ethiopia.
[9]Plate-Forme d'Infectiologie Expérimentale, PFIE, INRA, 37380, Nouzilly, France
[10]UCD School of Medicine, University College Dublin, Ireland.
[11]UCD School of Biomolecular and Biomedical Sciences, University College Dublin, Ireland.
[12]UCD Conway Institute of Biomolecular and Biomedical Science, University College Dublin, Ireland.

Current Addresses:
*Biomedical Sciences, Defence Science and Technology Laboratory, Salisbury, Wiltshire SP4 0JQ, UK.
§Kennedy Institute of Rheumatology, University of Oxford, Oxford OX3 7FY, UK.

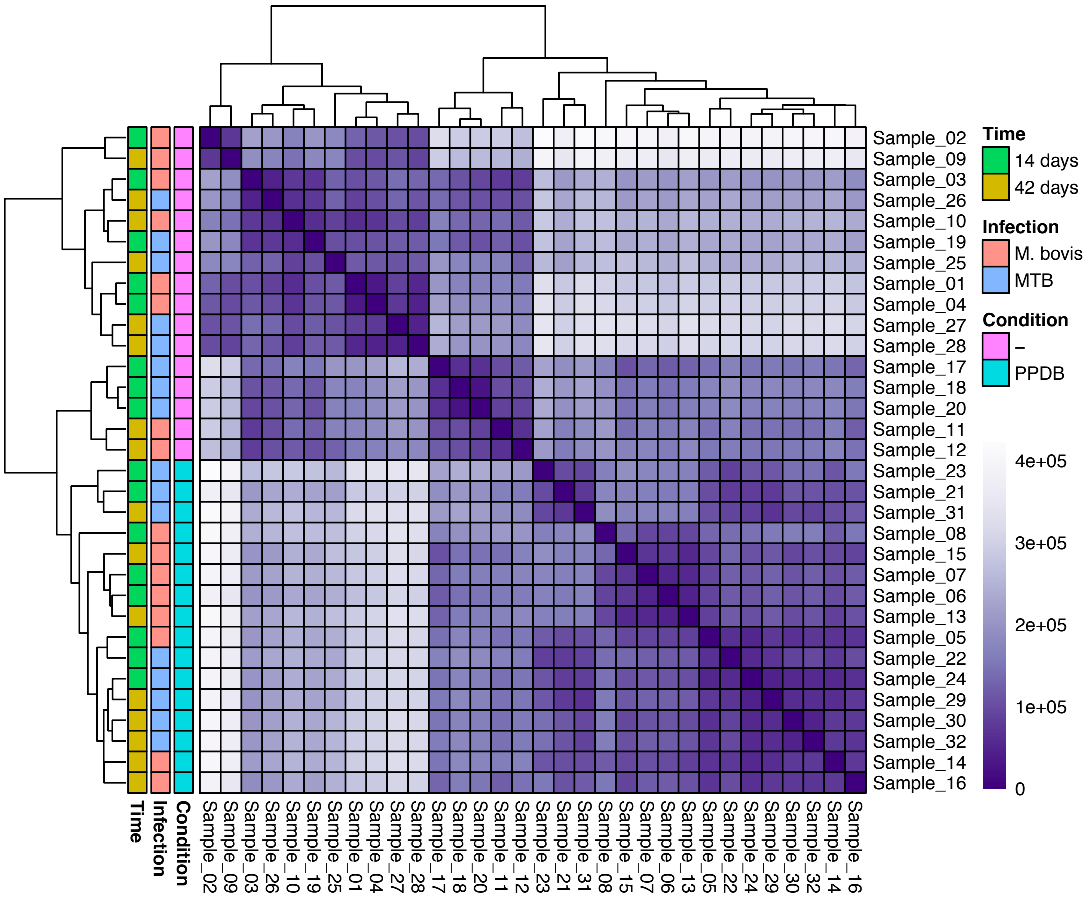

Figure S1 Genome-wide gene expression correlation
Genome-wide gene expression correlation between the 32 study samples pertaining to whole blood samples (unstimulated (“-“) or PPDb-stimulated (“PPDB”)) from cattle infected with either *M. bovis* AF2122/97 (“M. bovis”) or *M. tuberculosis* H37Rv (“MTB”) 14 days or 42 days (“Time”) post infection. Samples are clustered using the Euclidean distance and coloured bars on the left of the plot denote the variables time point (“Time”), infection status (“Infection”) and stimulation status (“Condition”) for each of the 32 samples.

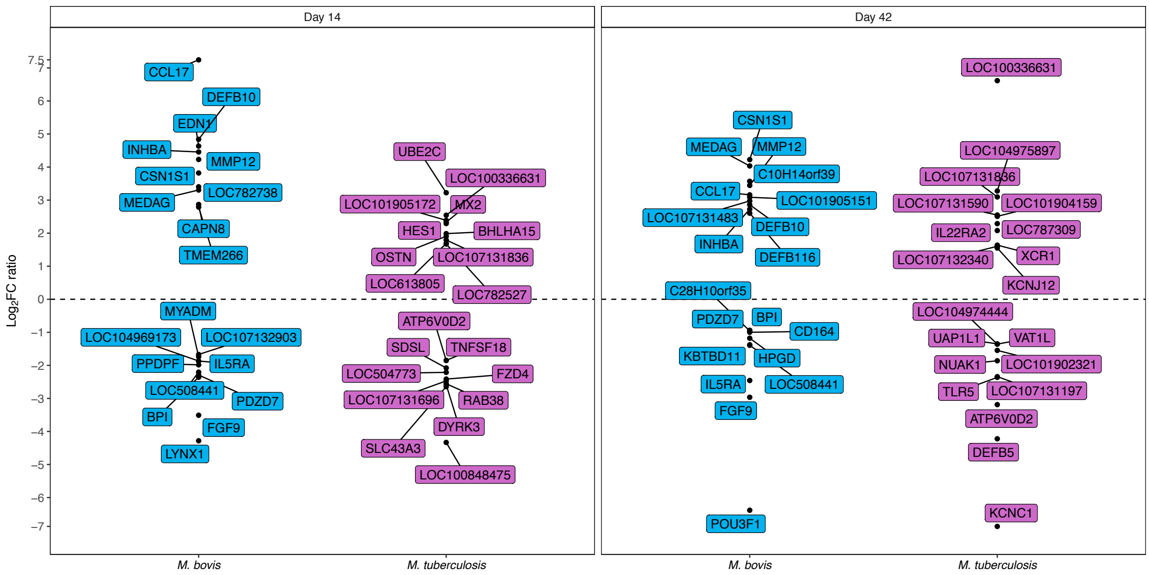

Figure S2 Top DE genes in unstimulated vs. stimulated whole blood between *M. bovis* AF2122/97 and *M. tuberculosis* H37Rv infected animals.

The top 10 upregulated and top 10 downregulated differentially expressed genes in whole blood samples derived from *M. bovis* AF2122/97 versus *M. tuberculosis* H37Rv infected animals and stimulated with PPD-B at day 14 and day 42 post infection. The change in gene expression from the comparison of stimulated blood to unstimulated blood at each time point for either *M. bovis* AF2122/97 or *M. tuberculosis* H37Rv infected animals was used to calculate $\log_2$FC ratio (i.e. expression of gene X in *M. bovis* AF2122/97 infected animals at day 14-post infection divided by expression of gene X in *M. tuberculosis* H37Rv infected animals at day 14-post infection).

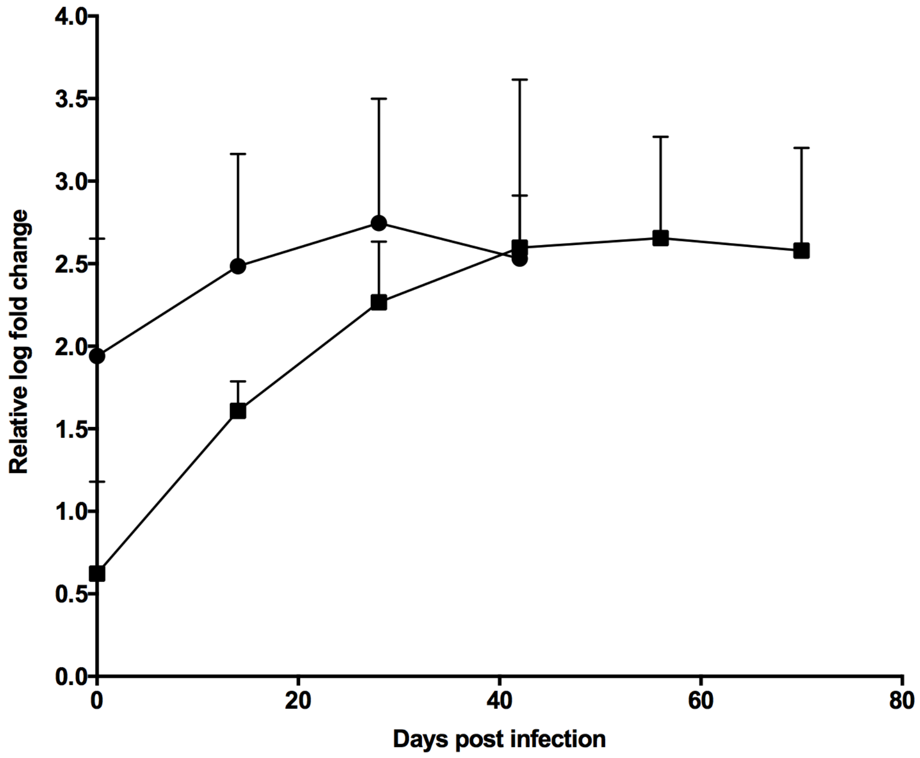

Fig S3 miR-155 analysis across *M. bovis* AF2122/97 and *M. tuberculosis* H37Rv infected animals.

The level of miR-155 in PPD-B stimulated vs. unstimulated whole blood from *M. bovis* AF2122/97 (circles) and *M. tuberculosis* H37Rv (squares) infected cattle was assessed over the infection time course using RT-qPCR with Exiqon miRCURY UniRT miRNA hsa-miR-155-5p primers.
